# Action, actor, context, target, time (AACTT): a framework for specifying behaviour

**DOI:** 10.1186/s13012-019-0951-x

**Published:** 2019-12-05

**Authors:** Justin Presseau, Nicola McCleary, Fabiana Lorencatto, Andrea M. Patey, Jeremy M. Grimshaw, Jill J. Francis

**Affiliations:** 1 Clinical Epidemiology, Ottawa Hospital Research Institute, Ottawa, Canada; 2 School of Epidemiology and Public Health, University of Ottawa, Ottawa, Canada; 3 School of Psychology, University of Ottawa, Ottawa, Canada; 4 Centre for Behaviour Change, University College London, London, UK; 5 Department of Medicine, University of Ottawa, Ottawa, Canada; 6 School of Health Sciences, City University of London, London, UK

**Keywords:** Behaviour, Framework, Behaviour specification, TACT, Behaviour change, Health professional behaviour

## Abstract

**Background:**

Designing implementation interventions to change the behaviour of healthcare providers and other professionals in the health system requires detailed specification of the behaviour(s) targeted for change to ensure alignment between intervention components and measured outcomes. Detailed behaviour specification can help to clarify evidence-practice gaps, clarify who needs to do what differently, identify modifiable barriers and enablers, design interventions to address these and ultimately provides an indicator of what to measure to evaluate an intervention’s effect on behaviour change. An existing behaviour specification framework proposes four domains (Target, Action, Context, Time; TACT), but insufficiently clarifies who is performing the behaviour (i.e. the Actor). Specifying the Actor is especially important in healthcare settings characterised by multiple behaviours performed by multiple different people. We propose and describe an extension and re-ordering of TACT to enhance its utility to implementation intervention designers, practitioners and trialists: the Action, Actor, Context, Target, Time (AACTT) framework. We aim to demonstrate its application across key steps of implementation research and to provide tools for its use in practice to clarify the behaviours of stakeholders across multiple levels of the healthcare system.

**Methods and results:**

We used French et al.’s four-step implementation process model to describe the potential applications of the AACTT framework for (a) clarifying who needs to do what differently, (b) identifying barriers and enablers, (c) selecting fit-for-purpose intervention strategies and components and (d) evaluating implementation interventions.

**Conclusions:**

Describing and detailing behaviour using the AACTT framework may help to enhance measurement of theoretical constructs, inform development of topic guides and questionnaires, enhance the design of implementation interventions and clarify outcome measurement for evaluating implementation interventions.

Contributions to the literature

- Behaviour change is a fundamental outcome of interest in implementation science and underpins change at multiple health care levels, but behaviour is often not clearly specified
- Detailed behaviour specification helps to clarify evidence-practice gaps, clarify who needs to do what differently, identify barriers and enablers, design interventions and provides an indicator of what to measure to evaluate an intervention’s effect on behaviour change
- Existing frameworks (TACT) do not clarify the Actor(s) (e.g. clinicians) performing the behaviour, an important distinction given multiple Actors and multiple Actions of interest in implementation science
- We propose an extension: the Action, Actor, Context, Target, Time (AACTT) framework for behaviour specification
- We provide practical tools to aid in AACTT-specification for a range of implementation study designs

## Background

Innovations in health research have the potential to improve health but harnessing this potential requires that effective innovations be translated into routine healthcare. Unfortunately, evidence-practice gaps continue to be documented: medicines are inappropriately prescribed [1], patient safety practices are not enacted [2] and harmful practices persist [3]. Sub-optimal clinical practices (over, under and misuse of tests/treatments) result in avoidable morbidity and mortality [4]. Such gaps in care are consistent across countries and clinical areas, leading some to suggest that health research is *‘*all breakthrough, no follow through’ [5]. Implementation science emerged in response to this, focusing on the rigorous scientific study and development of a cumulative evidence-base for how best to address evidence-practice gaps. A foundational requirement of implementation is the need for *someone* (usually more than one person or group), *somewhere* (from organisational leadership through to those providing direct patient care) doing *something* (usually more than one thing) *differently*. In short, taking up new evidence requires healthcare providers and other health system stakeholders to change their behaviour.

Given the centrality of behaviour in implementation science, there is a need for describing behaviour as clearly as possible. Doing so may help to (a) clarify evidence-practice gaps, (b) clarify the various people and groups at different levels that need to do something differently, (c) identify modifiable barriers and enablers and design implementation interventions to address them, (d) provide an indicator of what to measure to evaluate an intervention’s effect and (e) ultimately facilitate evidence synthesis. A generalisable framework may help to ensure consistency in the description and specification of behaviour in implementation research.

In the mid-twentieth century, after social psychologists identified that scores on attitude measures were not associated with actual behaviour [6], Fishbein [7] proposed that the low predictive validity of attitude measures could be addressed by assessing attitude to an *action* (e.g. *voting* for a specific political party) rather than assessing attitude to the *target* of that action (e.g. the political party). While now taken for granted, this focus on the *action* led to a paradigm shift in attitude-behaviour research and was a key principle underlying the Theory of Reasoned Action. Extending the approach, Ajzen [8] proposed the Theory of Planned Behaviour for predicting and explaining human behaviour in a specific context at a specific time. Together, these ideas gave rise to the specification of behaviour according to what became known as TACT: Target, Action, Context, Time [9]. Similarly, Michie and Johnston [10] proposed that when behaviours are described in terms of what, who, when, where and how, they are more actionable and hence more likely to be performed.

Clear specification of the behaviour is a key though often overlooked first step in conducting implementation research for a range of study objectives, such as identifying influences (barriers and enablers, and determinants) on behaviour or designing implementation interventions to support behaviour change among stakeholders in the health system. Despite half a century of guidance on behaviour specification, research is frequently published in which the behaviour is poorly specified. A systematic review of 67 reports of behaviour change interventions found that the Action domain was clearly specified in 69% of reports, and that all components of the TACT framework were described in only 5 (7.5%) reports [11]. Poor specification makes it difficult to measure behaviour and behaviour change. Clear specification facilitates strong compatibility between the behaviour under investigation and the theoretical constructs that predict that behaviour, which enhances prediction (cf. the principle of compatibility) [12, 13].

Consider the following description of a potential guideline-recommended clinical behaviour for primary care practitioners: ‘For people with diabetes, record a blood pressure reading in the patient’s medical records’ (Example 1). Using the TACT framework to unpack this recommendation, the two specified components are the *Target* (people with diabetes) and the *Action* (record a blood pressure reading in the patient’s medical record). While a seemingly straightforward description, it is not clear who should do the behaviour and when and where it should take place. Leaving this implicit introduces ambiguity that may undermine change efforts as well as measurement of whether or not the behaviour has been performed.

Drawing upon Fishbein and Ajzen’s [9] early advice, a clearer, more actionable specification could be: ‘For people with diabetes (*Target*), record a blood pressure reading in the patient’s medical records (*Action*) in the primary care clinic (*Context*) when they attend for their annual diabetes review (*Time*)’ (Example 2). Example 2 specifies further components of the behaviour: Time (when patients attend) and Context (primary care clinic). This enhanced specification corresponds to the TACT framework, but still lacks a fundamental component: *who* (i.e. which person or people on the primary care team) is responsible for performing the Action. Furthermore, Example 2 arguably involves a sequence of discrete Actions (taking the blood pressure reading, accessing the patient’s medical records, entering the blood pressure reading into the record). It may also include ancillary behaviours such as inviting the patient to attend the clinic for the annual review. These actions may be performed by different primary care staff (e.g. physician, nurse, administrator) to support the focal behaviour of interest. This dimension of specification is not included in the TACT framework, which assumes that the individual is performing the behaviour for themselves. In implementation research, individuals often perform a behaviour for someone else’s benefit (i.e. the Target, such as a patient). We propose to expand the TACT framework to guide specification of behaviour in terms of not only Target, Action, Context and Time but also Actor—the person(s) who will perform the Action(s). By clarifying the Actor, the Action then becomes clearer and more specific, allowing for clarification of complex behaviours (or sequences of behaviour) in terms of different Actions performed by different Actors in the health care setting at different times (i.e. preparatory and sequential Actions).

### AACTT: an expanded framework for specifying behaviour

We propose the AACTT framework (Action, Actor, Context, Target, Time) for specifying behaviour. Re-arranging the order of domains in the framework reflects a more easily defined sequence for specifying behaviour than TACT that naturally begins with the Action and who performs it.

Although it may sometimes seem obvious who is to perform the Action, for behaviours that are performed by healthcare professionals or teams for, with or on behalf of their patients, specification of the Actor is particularly helpful. Indeed, healthcare delivery behaviours have been described as ‘collective behaviours’, suggesting that role confusion may be a barrier to performance [14] that could be illuminated by careful specification of the behaviour at the outset using AACTT.

While Action and Actor are important, specification of Context and Time allows the responder to keep these elements in mind when answering a questionnaire, responding in an interview or changing their behaviour. Behaviours are inherently tied to the time and place in which they occur, and thus clarification of these elements provides an opportunity to situate analyses of barriers and enablers and intervention design within the contexts that behaviours take place. Recent theoretical advances emphasise the role of associative processes in behaviour [15], including automatic processes [16–19]. Specification of contextual and temporal cues in questionnaire items, interview topic guides and observational tools may increase the validity of responses, especially if the behaviour has an element of automaticity. Context and time may also be important for identifying when and where it is appropriate to perform an Action, thus informing implementation efforts.

When considering the behaviour of individuals and teams as they deliver health care, a further refinement of the ‘Target’ domain is appropriate. ‘Target’ is often explained as (performing) an Action *to* someone, i.e. who the behaviour is targeted at. However, current models of healthcare delivery place a focus on patients as active participants in their health care, and thus as a collaborator with the healthcare professional. Hence, rather than performing an Action *to* a passive recipient, the healthcare professional may act *with* or *for* the patient. Thus, it is recommended that researchers frame (i) Action and (ii) Target as (i) doing what? (ii) *to*, *for*, *with* or *on behalf of* whom?

In doing so, the Actor-Target relationship need not only reflect a healthcare professional-patient relationship. As demonstrated in Fig. 3, for healthcare professionals working in a team, one healthcare professional’s (Actor A) behaviour (Action A) may be for the benefit of another healthcare professional (Target A), enabling the latter’s subsequent behaviour (Action B). Such horizontal sequences within teams also apply to specifying the behaviour of vertical sequences of behaviour within the health system, where a policymaker’s (Actor A) behaviour (Action A) sets the stage for a healthcare administrator (Target A/Actor B) to perform a behaviour within their role (Action B) that benefits the healthcare professional (Target B/Actor C) and enables them to provide care (Behaviour C) to benefit their patients (Target C). Thus, a given Actor’s Target can also be another Actor in the system.

AACTT provides common elements that can be used for consistent description and specification of behaviour. By extension, AACTT can be used to describe the sequence of multiple behaviours of multiple Actors at different levels of the organisation required to enact change. For instance, in the case of promoting hand hygiene in hospital, AACTT provides a means for clarifying the behaviour of those engaging in hand sanitizing behaviour, but also the leadership in the organisation whose policy-enacting behaviour sets the stage for middle management to engage in procurement behaviour to provide hand sanitizing stations and gels, through to maintenance staff engaging in refilling behaviour to ensure sanitizing gel is available for the healthcare providers. Each behaviour by these organisational Actors is required for healthcare providers to engage in hand-sanitizing activities. Rather than making implicit assumptions about such a sequence of behaviours or describing them as separate organisational factors, the AACTT framework helps to unpack the complexity and clarify the responsibility of all behaviours in organisational health settings, providing a clear opportunity for behavioural approaches to inform organisational change. The level of granularity or aggregation in the specification of each AACTT domain should be defined by what is measurable, useful and practical for the given application, to ensure practical utility.

In summary, we propose a new framework: AACTT (Action, Actor, Context, Target, Time—see Table 1 for definitions and examples) to allow for the careful delineation of ‘who does what; to, for or with whom; when; where?’ [10]. Herein, our aim is to demonstrate how the AACTT framework can be used within the main steps of an implementation research process and to provide a simple tool that implementation researchers and practitioners can use to apply the AACTT framework to specify the behaviour(s) of stakeholders.

**Table 1:** AACTT framework definitions and examples

AACTT domains	Definition^1^	Examples
Action	A discrete observable behaviour	Prescribing antihypertensives, providing a referral to a specialist, washing hands, setting a policy
Actor	The individual or group of individuals who perform (or should/could) the Action	Primary care physician, pharmacist, social worker, resident, administrator, middle manager, head of unit, policymaker
Context	The physical, emotional or social setting in which the Actor performs (or should/could) the Action	Examination room, doctor’s office, outside a patient room, in a boardroom, stressful vs. calm situation, when patients’ relatives are present or not
Target	The individual or group of individuals for/with/on behalf of whom the Actor performs the Action	Patient with diabetes and blood pressure above 140/80 mmHg, patient wanting to quit smoking
Time	The time period and duration that the Actor performs the Action in the Context with/for the Target	At annual review, next time a patient visits, every week, over the next 6 months

^1^ from Francis et al (2004)

## Methods

We used French’s four-step implementation process model [20] to exemplify how AACTT can be applied to each of the key steps of the implementation process: Step 1 (clarifying who needs to do what differently), Step 2 (using a theoretical framework, which barriers and enablers need to be addressed), Step 3 (which intervention components could overcome the modifiable barriers and enhance the enablers) and Step 4 (how can behaviour change be measured and understood). We selected French’s process model to guide the demonstration of AACTT’s application given that its foundational step involves clarifying the behaviour. However, AACTT aligns with other process models (e.g. Knowledge-to-Action Framework [21], the UK MRC framework for developing and evaluating complex interventions [22] and process evaluations [23]). At each step, we identified how the AACTT framework could be applied and provide published examples across a range of methodological approaches which align with those commonly used in implementation research [24].

## Results

### Step 1—Using AACTT to identify who needs to do what, differently, when and where

**Fig. 1:**
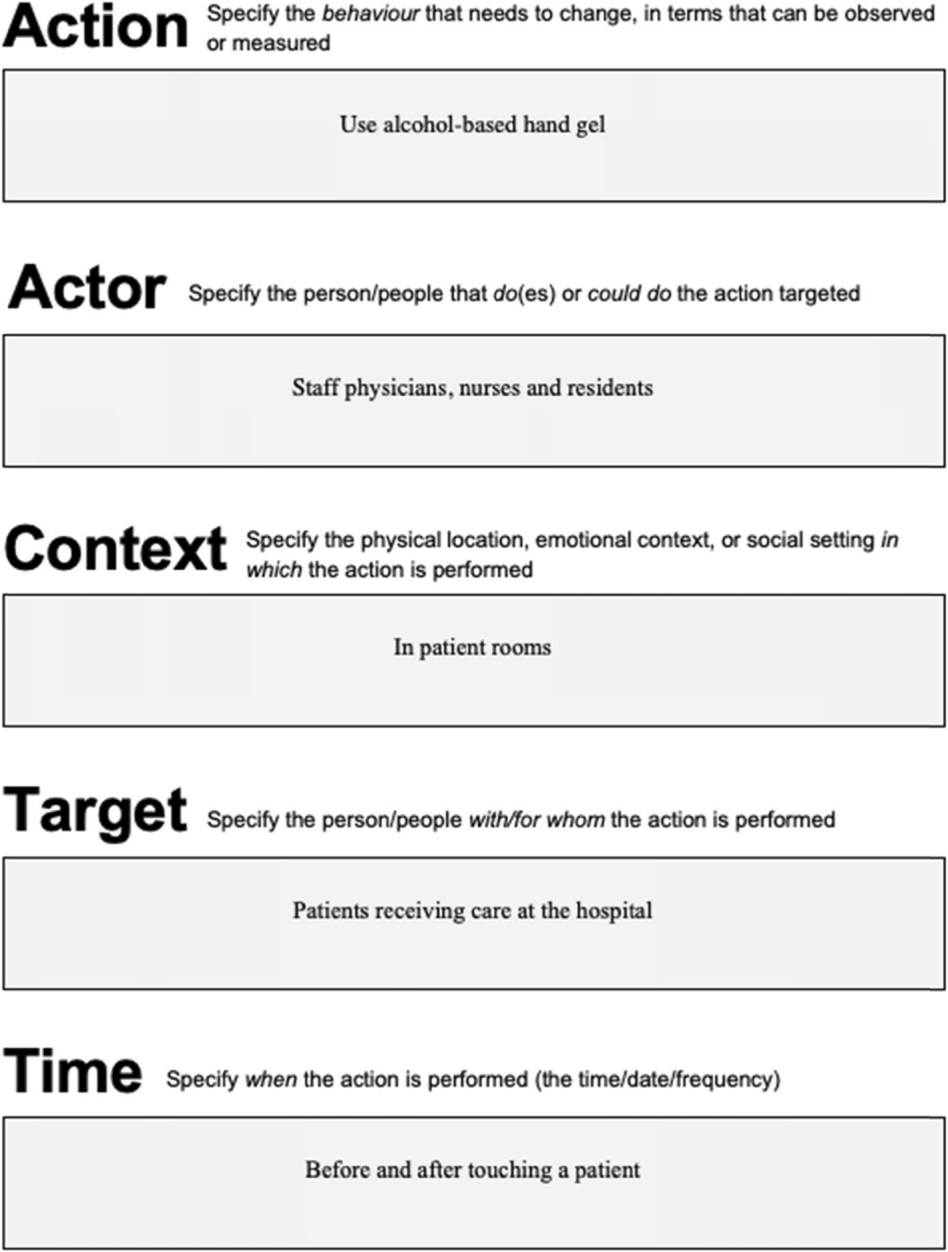
AACTT specification tool for a single Action, with worked example applied to improving hand hygiene

**Fig. 2:**
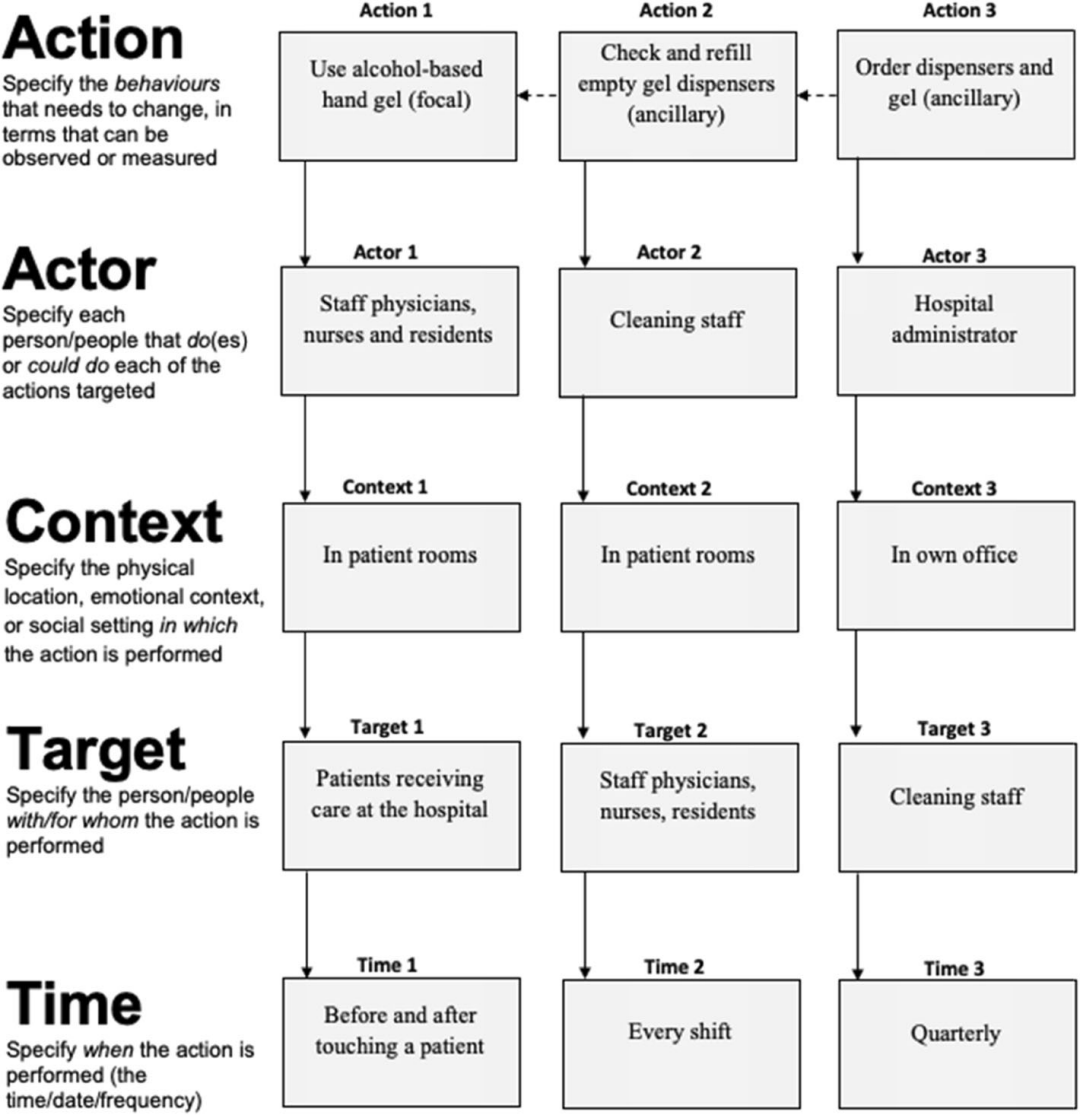
AACTT specification for focal and ancillary Actions of multiple Actors, Contexts and Times, with worked example applied to improving hand hygiene

**Fig. 3:**
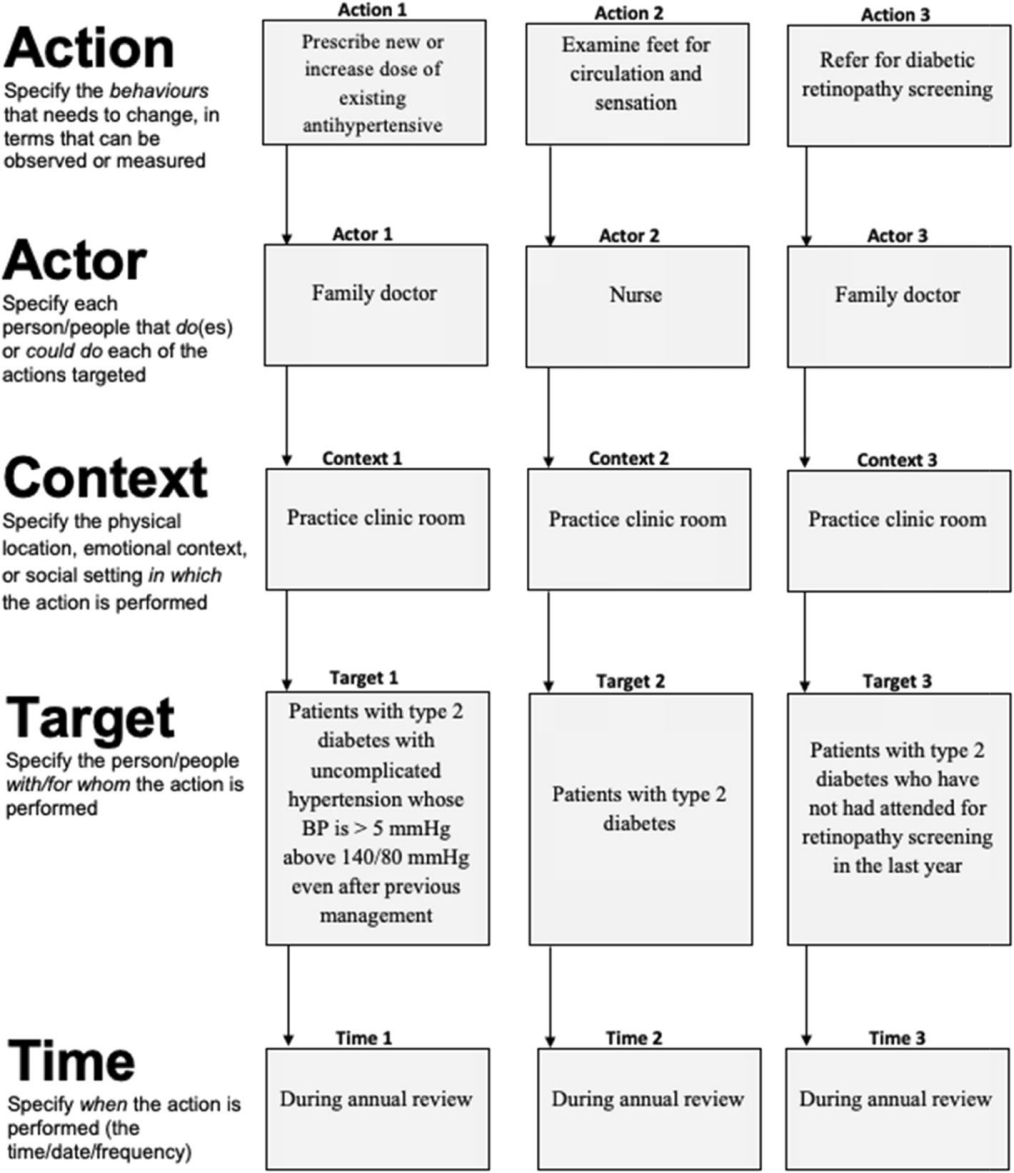
AACTT specification tool for team-based Actions and Actors with variable Target and consistent Context and Time, with worked examples applied to diabetes care

The AACTT framework can be used at this foundational step of implementation research to ensure coverage across key behaviours (Actions), stakeholders (Actors, Targets) in the particular settings (Time, Context) of desired performance. This helps ensure that subsequent steps remain consistent with the AACTT-specified behaviour(s) established at the outset. Application of the AACTT framework at this step can help to identify which individuals at which levels of an organisational hierarchy need to do something differently to implement an evidence-based practice (or indeed de-implement an outdated or non-evidenced practice). Actors often include healthcare professionals, but may also involve their colleagues within a team, administrative staff and middle and upper management whose own Actions facilitate the clinical behaviour of the health professional, and ultimately patients and citizens behaviour. One can extend Actors to people at multiple levels in a healthcare system, e.g. organisational leadership, policy makers and political Actors, whose own behaviours may be centrally important to ensuring that the health professional can engage in a given guideline-recommended behaviour under investigation. The AACTT framework can be employed to unpack complex organisational and hierarchical inter-relationships into the multiple observable, measurable (and changeable) Actions of individuals at each level. This can provide the basis for clarifying whose behaviour to focus on in a given investigation. Figures 1, 2 and 3 provide generalisable worksheets with a worked example that can be used by implementation researchers and practitioners, on their own or as part of a stakeholder engagement activity, to clarify who needs to do what, differently (see also Additional file 1 for blank versions of each worksheet).

The implication of identifying who needs to do what differently also suggests a need to clarify a gap in care. In specifying behaviour using the AACTT framework, it may become clearer where specifically evidence-practice gaps exist (i.e. specifically whose behaviour in what settings and time) rather than broader generalisations of evidence of gaps in care typically available to justify a focused implementation diagnostic effort.

### Step 2—Using the AACTT framework to inform investigations to identify barriers and enablers

Having an AACTT-specified behaviour(s) can inform more focused investigations into the barriers and drivers of Actions for each Actor. A key benefit to specifying the Actor using this framework is that it helps to identify which specific agents one should engage with in eliciting their barriers and enablers to performing the Action; focusing consideration down specifically on those who are or could perform the Action. This may help in qualitative and quantitative approaches to investigating barriers and enablers, including interviews/focus groups to generate qualitative data and questionnaire-based quantitative data operationalizing theoretical constructs hypothesised to correlate with implementation behaviour. At the initial stages of inquiry, it may not yet be clear who all these potential Actors and Actions are but AACTT specification allows this to be further clarified and be made explicit. Initial broad specification at a higher level (e.g. primary care staff including nurses and physicians) may help to recognise the potential for multiple Actors to undertake the same Action, and to explore during the barrier elicitation whether this diffusion of responsibility is problematic (and thus may benefit from greater specification for each Actor to promote role clarity) or acceptable/desirable (in which case a given setting may find it useful to allocate clear responsibility).

#### Interviews/focus groups

An AACTT-specified behaviour provides greater focus to an interview or focus group to maximise the likelihood that responses are reflective of the specific Actions of the Actors situated in a time and place, which may also help with recall. Specifying the Actor up front helps to inform recruitment so that the respondents are those actually tasked with the Action (as opposed to others speaking on their behalf). Topic guides can then be designed to ensure that interview and focus group prompts are consistently focused on the AACTT-specified behaviours of the respondents (Actors). For instance, a broadly specified behaviour such as ‘improving blood pressure prescribing for patients with type 2 diabetes’ lacks AACTT-specificity and thus interview/focus group questions aiming to unpack views on readiness to change [25], beliefs about consequences and social influences [26, 27] or implementation climate and culture [28] may remain at a level that does not clarify how those views translate into understanding who needs to do what differently and why they do or do not. In contrast, while ‘increasing the dose of existing antihypertensive medication (Action) by a family physician (Actor) during annual diabetes review (Context) for their patients with type 2 diabetes (Target) when their blood pressure is above 140/80 mmHg despite previous management (Time)’ provides an admittedly longer description, the specificity allows much greater insight into the factors that may determine this behaviour than might otherwise be missed by a broader specification. Describing the AACTT upfront also allows similar topic guides to be tailored to different Actors who may be engaging in the same or different Actions.

This is particularly aided when using theoretical frameworks to inform interview topic guide development, such as when using the Theoretical Domains Framework [26, 27], COM-B within the Behaviour Change Wheel [29] or Consolidated Framework for Implementation Research [28, 30, 31] to elicit views about barriers and enablers that are rooted in a given domain or construct. An AACTT-specified behaviour can also help a directed content analysis of theory-informed interviews by providing the capacity to code barriers and enablers described by respondents directly linked to a given AACTT-specified behaviour, to ensure that the barriers identified are in fact related to the Actions and Actors of interest. Importantly, AACTT-specification ensures that barriers and enablers are specifically those linked to a particular Actor and behaviour in the time and context of performance, rather than vague and broad barriers/enablers. This facilitates more targeted intervention development in Step 3.

#### Questionnaires

The original TACT framework was established to inform the careful development of questionnaire items to operationalise constructs from the Theory of Reasoned Action, and then the Theory of Planned Behaviour that were used to predict behaviour using cross-sectional and prospective designs. This approach, known as the principle of compatibility, involves developing questionnaire items tailored to the specific Target, Action, Context and Time to ensure consistency between the items measuring theoretical constructs and the subsequent behaviour being predicted while ensuring rigour in operationalisation of the theoretical constructs. The TACT-informed principle of compatibility was widely adopted in the 1980s and 1990s when testing of the Theory of Reasoned Action and Theory of Planned Behaviour grew in popularity in social and health psychology. At the turn of the century, these social cognition models began to be adapted and adopted for use in implementation research to predict the behaviour of healthcare professionals. Francis and colleagues developed specific guidance for developing Theory of Planned Behaviour-based questionnaires for use to understand and predict health care professional behaviour [32]. This highly cited and influential guidance provided a description of how the TACT framework could be applied in this setting and influenced subsequent rigorous development of Theory of Planned Behaviour questionnaires to understand how its constructs explain variability in the behaviour of health care professionals (see Godin et al 2008 for a review [33]). Such methods were also then adopted to operationalise other theories, while to date this has largely been confined to theories of behaviour [34], there is an opportunity to consider how AACTT-specification can be integrated into the operationalisation of questionnaire items for other theories, models and frameworks used in implementation science and in particular those for which there is concerted effort to developed measures such as Consolidated Framework for Implementation Research [35], Organizational Readiness for Change [36] and Normalization Process Theory [37]. Huijg and colleagues ([38], see their Table 5) developed and established the discriminant content validity of questionnaire items designed to assess domains from the Theoretical Domains Framework in such a way to allow any Action, Actor, Context, Target, Time to be integrated into the measure, and Eccles and colleagues operationalized questionnaires consistent with AACTT for a range of theories of behaviour ([34] see the Additional file 2 in Huijg et al. for examples of the questionnaire items that they developed). Opportunities present themselves for similar adaptations for other models, theories and frameworks.

### Step 3—Using AACTT to inform selecting and operationalizing implementation intervention components to address barriers and enablers

Best practice in implementation intervention development involves selecting evidence-based techniques and strategies that address the barriers and enablers identified [39], and clarifying who delivers and receives the intervention, how, when, where and how much [40]. Implementation intervention developers have a range of tools at their disposal for selecting and specifying interventions: high-level descriptors of implementation strategies such as those described in Cochrane’s Effective Practice and Organisation of Care (EPOC) [41] and Expert Recommendation for Implementing Change (ERIC) [42] taxonomies and the Behaviour Change Wheel [29], as well as fine-grained descriptors of techniques such as those proposed in Behaviour Change Techniques Taxonomy [43], Intervention Mapping [44] or emerging tools for linking behaviour change techniques to mechanisms of action [45]. Regardless of the strategies and techniques, it can help to know specifically whose (Actor) behaviour (Action) is being targeted for change with the strategies/techniques, and where (Context) and when (Time) the behaviour is expected to be performed, for/with whom (Target). This may help in narrowing and prioritizing amongst potential strategies and techniques. By having AACTT-specified behaviours defined, many of these decisions are made clearer and help to operationalise the strategies themselves. For instance, clearly articulated AACTT-specified behaviour can (i) inform selection of performance indicators provided to primary care physicians as part of an Audit and Feedback (A&F) intervention, (ii) ensure that the A&F intervention is directed to the correct Actors and (iii) that it reflects the context and times of performance and (iv) that the data reflect the Target patients.

A multicomponent intervention involving multiple behaviours and Actors can also be aided by AACTT specification. For instance in a hospital setting, an intervention to improve hand hygiene may involve the focal behaviour of sanitizing hands using alcohol-based gel (Action) performed by nursing staff, surgeons, residents (Actors), as well as ancillary behaviours such as purchasing hand sanitiser (by administrators) and checking/refilling dispensers (by cleaning staff) that are necessary in supporting the focal behaviour. Other settings, such as primary care, might similarly involve a focal Action of initiating a new medication or deprescribing another as well as ancillary Actions, each with respective Actors (e.g. family doctors, nurse practitioners, nurses, administrative staff, practice managers). Community settings could also involve other professional groups (e.g. social workers), parents/carers and teachers. For example, regarding implementing dietary menu guidelines in day care (nursery) services [46], the action could be ‘preparing food consistent with childhood dietary guidelines’, the actor could be ‘cooks’, the context ‘in the day care centre kitchen’, the target ‘children attending day care’ and the time ‘at lunch every week day’. Regardless of the apparent complexity of the implementation intervention being developed, the AACTT framework can provide transparency and clarity in terms of whose behaviour is targeted by the strategies in the intervention.

### Step 4—Using AACTT to specify how behaviour change can be measured and understood

The application of the AACTT framework in previous steps ensures compatibility with the measures used to assess change in Step 4. Implementation researchers are often interested in both whether an intervention changes behaviour (outcome) as well as explaining the (theory-informed) mechanisms through which change occurs.

#### Intervention outcome evaluation

Irrespective of the design used to evaluate an implementation intervention, a measure or indicator of behaviour is often central to demonstrating change. In some instances, outcome measurement is embedded within available routinely collected data, such as prescribing, ordering and referring, or specific process data collected locally or as part of larger scale initiatives (e.g. Quality and Outcomes Framework in the UK) and national audits. When such data are accessible, they provide a pragmatic, low-burden means of evaluating implementation interventions. However, this often involves having to balance consideration of pragmatism with those of measurement specificity. While the likelihood is low that routinely collected data perfectly correspond with the Action performed by the targeted Actor in the Context and Time for the Target designed for the intervention, having an AACTT-specification allows clarity of the degree (or not) of this correspondence. While it is true that in randomised designs, any additional ‘noise’ introduced by pragmatic outcome measures would at least be balanced by the randomisation, the more ‘noise’ (i.e. error variance) the less power to detect change in the actual outcome of interest, which has implication for sample size calculations. Thus, even in randomised designs, AACTT-specification provides an opportunity to establish the degree of correspondence between the targeted behaviour(s) and the indicators of behaviour available to assess the degree of noise (error) in the outcome.

In evaluations where no routinely collected data are available, outcome measures are sometimes developed for the intervention evaluation itself. In such instances, the added advantage of AACTT specification is that it can directly guide which data to collect and provides full control over what ‘counts’ as performance both in terms of the behaviour of interest as well as ancillary behaviours that necessarily support the behaviour of interest but in themselves are not sufficient.

#### Intervention process evaluation

In addition to evaluating whether an implementation intervention is effective, it is important to understand the mechanisms through which this effect occurred both in terms of changing the targeted mediating constructs (mechanisms of change) and assessing delivery and receipt as designed (fidelity). AACTT-specified behaviours provide the same advantage as in Step 2 for informing the design of qualitative or quantitative assessment of mechanisms of change alongside outcome assessment, such as in theory-based process evaluations [47–49]. Using AACTT to inform the wording of process measures that operationalise the theoretical constructs targeted for change provides measures of mediators tied directly to the behaviour the intervention is targeting for change. This provides more direct correspondence between mechanism and outcomes. In this instance, AACTT-specification provides greater measurement sensitivity.

AACTT-specification also allows for more careful assessment of fidelity (and adaptation) of delivery and receipt of an implementation intervention by providing structure and transparency in terms of who should receive the intervention (Actor) and which Action(s) under which circumstances (Time, Context) for which Targets. This specificity and transparency can clarify what and whom to track to assess fidelity and adaptation. Table 2 provides worked examples of AACTT specifications across study designs.

**Table 2:** Worked examples of AACTT-specified behaviours across study designs

Study design	Interviews (qualitative) [55]	Questionnaire (quantitative) [34]	Intervention development [56]	Cluster randomised trial [57]	Fidelity assessment (process evaluation) [58]	Mechanism of change (process evaluation) [59]
Action	Managing back pain without X-ray	Prescribing additional antihypertensive drugs	Provision of sexual counselling group sessions	Examining feet yourself and/or referring for foot exam	Providing behavioural support for smoking cessation (detailed by component behaviour change techniques)	Advising patient to make an appointment for retinal screening within the next 12 months
Actor	Chiropractors	General practitioners	Cardiac rehabilitation healthcare staff	General practitioner and nurse	Trained specialist stop smoking advisor	Family doctors
Context	Private clinics (Canada, USA with an HMO)	Practice clinic	Hospitals in the Republic of Ireland	Practice clinic	English stop-smoking service clinic in East London and North England	Examination room in family practice
Target	Patient with acute low back pain	Patients with type 2 diabetes whose blood pressure (BP) is 5 mmHg above a target of 140 mmHg systolic BP or 80 mmHg diastolic BP even following previous management	Patients aged 18+ with cardiovascular disease	Patients with type 2 diabetes	Smokers trying to quit	Specific patient scenario: 57-year-old woman with type 2 diabetes on metformin 500 mg, non-smoker, no other medication, A1C < 7%, BMI 25, BP 125/75, normal foot exam, attended retinal screening over 12 months ago
Time	During patient visit	Over the next 12 months	During Phase III cardiac rehabilitation	In the last 12 months	Over four weekly sessions	During annual diabetes checkup

## Discussion

Herein, we introduced the AACTT framework that can be used to inform the careful specification of behaviour for implementation research and practice and provided a generalisable worksheet to facilitate use of the framework (worked examples in Figs. 1, 2 and 3, blank worksheets in Additional file 1). AACTT can be applied across key steps of implementation research and practice advocated by process models [20–22] to transparently define and measure behaviour(s) in terms of who performs them, for/with whom, when and where. AACTT formalises a natural progression of the TACT framework developed by Fishbein and popularised in social and health psychology for more direct application to behaviour where the individual is performing a behaviour for someone else’s (i.e. Target) benefit. This has direct application not only in implementation science applied to healthcare settings but also public health–, social welfare– and family-based settings in which someone performs an Action for someone else (e.g. school- and family-focused interventions) [46, 50].

Using the Theory Comparison and Selection Tool (T-CaST) and checklist [51], the AACTT framework is designed to be *usable* (includes relevant domains, has been developed so that key stakeholders can use it, we provide steps for its application and methods for promoting its application across a range of possible studies and an explanation for how the domains influence each other), *testable/valid* (can form the basis for testable hypotheses, includes face-valid explanations and has been used in empirical studies), *applicable* (focuses on a key implementation outcome, can be applied across a range of methods and across a range of analytical levels, populations and conditions and is generalisable across disciplines) and is likely to be *acceptable* (to key stakeholders, and is the historical evolution of a framework rooted in a particular discipline). Thus, in principle it fulfils all the criteria for use of a framework by implementation science researchers and practitioners, though its actual usability, testability, applicability and acceptability will ultimately be determined through application of the tool across a range of types of implementation research [52].

Michie and Johnston made a call for making clinical practice guidelines more specific by specifying ‘what’, ‘who’, ‘when’, ‘where’ and ‘how’ [10]. While sharing some similarities with AACTT, there are three important differences that distinguish AACTT and underscore its potential added utility. First, the ‘who’ in Michie and Johnston’s recommendations refers to the ‘Actor’ but does not make any mention of ‘to, for or with whom’ the action is performed (i.e. the ‘Target’ in AACTT, which may be a patient, a healthcare team member or other organisational actor). Second, the ‘where’ is specific to a physical location, whereas ‘Context’ in AACTT can refer to a broader set of contexts that include the physical location but could also include a context that is internal to the actor (e.g. emotional context of a stressful versus a calm situation) or the social context (e.g. when patients’ relatives are present). Third, in proposing AACTT as an extension to Fishbein and Ajzen’s TACT framework, we are deliberate in ensuring cumulative theory and methods development as it extends to applications within implementation science.

Existing calls for better specification and reporting have focused on the description and labelling of implementation intervention strategies (e.g. with taxonomies of change strategies such as the ERIC [42] and BCT [43] taxonomies) and on the wider components of interventions (e.g. with checklists such as TIDIER [40]). Within implementation science, Proctor, Powell and McMillen [53] proposed seven domains for specifying implementation strategies per se, including who delivers the strategy (actors), how they deliver the strategy (actions), what and to whom the strategy is directed (action target), the sequence of strategy delivery (temporality), intensity (dose), implementation outcomes affected and justification. While some of the Proctor et al. domains share similar or overlapping nomenclature with AACTT (i.e. Actor, Action, Time), the scope of intended application of each framework and its domains differs. As exemplified in Table 1, in the AACTT framework ‘Actor’ refers to the individuals or groups performing the behaviour that could be the recipient of an implementation strategy, the ‘Action’ refers specifically to the behaviour being performed by the Actor and is the object of change and ‘Time’ refers to when that behaviour is performed. Thus, whereas the seven dimensions proposed by Proctor et al. focus on detailing the delivery of an *implementation strategy* within an implementation intervention, AACTT domains focus uniquely on detailing *behaviour(s)*. Thus, in the context of an implementation intervention that would be specified with Proctor et al.’s dimensions, the behaviour specified using AACTT would be an outcome that the (now well-specified) implementation strategy aims to change. Thus, AACTT is designed for specifying behaviour as the object of change, rather than specifying implementation strategies designed to bring about that change.

## Conclusion

The careful description of outcomes and measures is of central importance to the development of a cumulative and generalisable science. The AACTT framework provides a means for establishing the core elements of behaviour targeted for change. The framework can be applied to describe the behaviour of Actors at multiple organisational levels. The framework facilitates the systematic description of who needs to do what differently, understanding what may stop or help them from doing so, how to support them to address barriers to change and how to demonstrate that such support worked. AACTT is compatible with any theory, model or framework in which behaviour is the focus of inquiry and thus has the potential to be integrated alongside key theoretical approaches used in the field. Given on-going efforts to systematise the measurement of theoretical constructs [54], and description of intervention components [41–43], there is a need for ensuring that the object of change, i.e. behaviour, is equally as carefully described and understood. This will ultimately contribute to a greater capacity to synthesise research over time.

## Supplementary information

**Additional file 1.** Blank worksheets for single-actor and multiple-actor/action AACTT specification.

## Data Availability

Not applicable.

## Abbreviations

AACTT: Action, Actor, Context, Target, Time
A&F: Audit and Feedback
COM-B: Capability, opportunity, motivation, behaviour
EPOC: Effective Practice and Organisation of Care
ERIC: Expert Recommendation for Implementing Change
TACT: Target, Action, Context, Time
T-CaST: Theory Comparison and Selection Tool
