# Supplementary material for: Action, actor, context, target, time (AACTT): a framework for specifying behaviour
Source: Implement Sci. 2019 Dec 5;14:102. doi: 10.1186/s13012-019-0951-x (PMC6896730; doi:10.1186/s13012-019-0951-x)
Supplement: Supplementary file 1 — Additional file 1. Blank worksheets for single-actor and multiple-actor/action AACTT specification. [file 13012_2019_951_MOESM1_ESM.pdf]

Figure 1. Tool for single-actor AACTT-specification

Action

Specify the *behaviour* that needs to change, in terms that can be observed or measured

Actor

Specify the person/people that *do*(es) or *could do* the action targeted

Context

Specify the physical location, emotional context, or social setting *in which* the action is performed

Target

Specify the person/people *with/for whom* the action is performed

Time

Specify *when* the action is performed (the time/date/frequency)

Figure 2. Tool for multiple-actor/action AACTT-specification

Action

Specify the *behaviours* that needs to change, in terms that can be observed or measured[1,2]

Actor

Specify each person/people that *do*(es) or *could do* each of the actions targeted[2,3]

Context

Specify the physical location, emotional context, or social setting *in which* the action is performed

Target

Specify the person/people *with/for whom* the action is performed[4]

Time

Specify *when* the action is performed (the time/date/frequency)

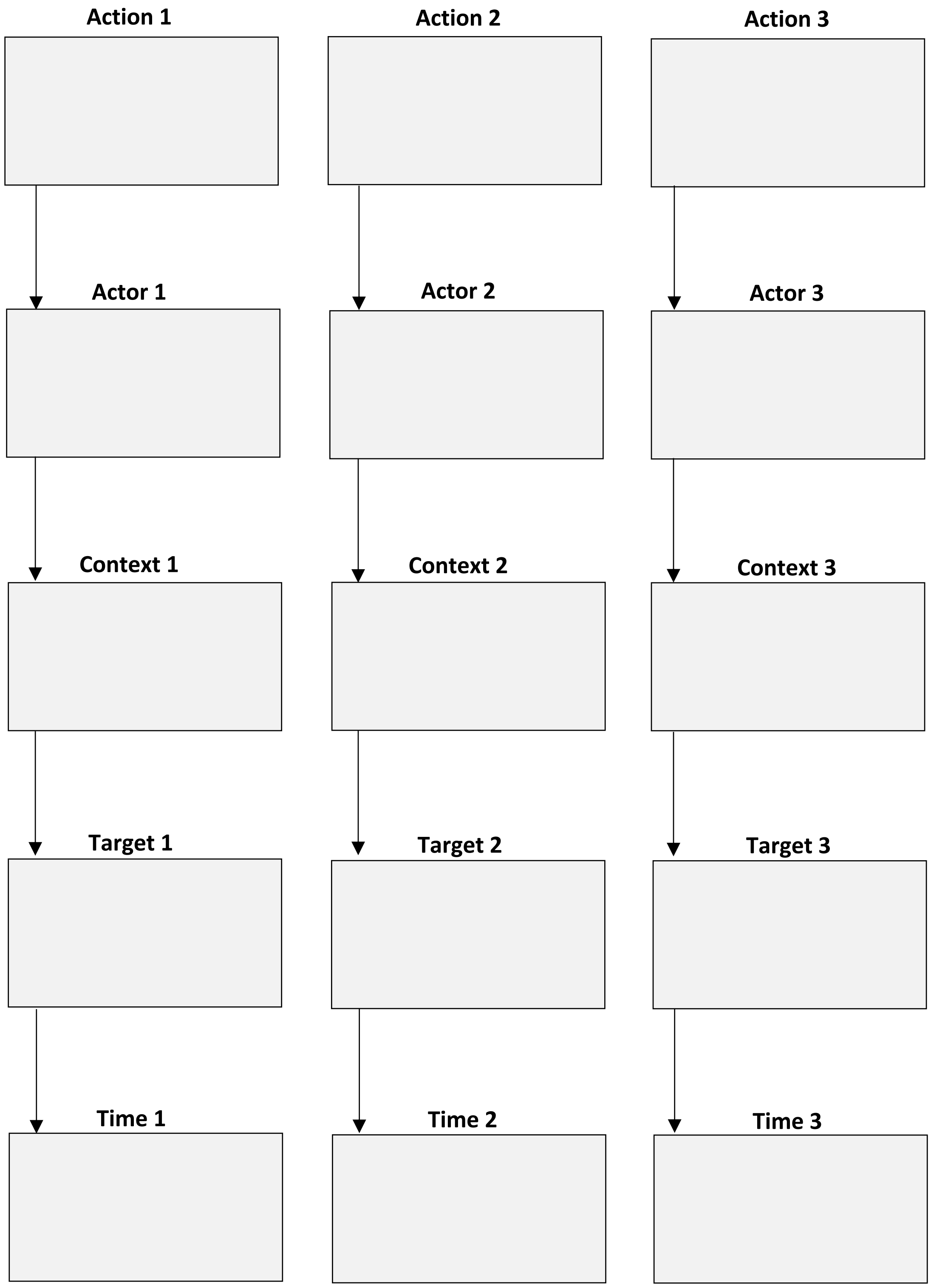

[1] Actions can be either a behaviour of the key actor of interest or an ancillary behaviour that supports another actor and action.
[2] Add arrows between behaviours when one supports another.
[3] Actors can be anyone at any organizational level (e.g. healthcare professional, patient, member of the public, teacher, family member, middle manager, leader)
[4] Actors can be different members of a team/group that work together in the same context and time or could be linked organizationally so that e.g. behaviour of a leader provides necessary capacity for a healthcare professional's behaviour
[5] For healthcare providers, the target might often be a patient, but could be colleague. For leaders, middle managers, the target would often be the healthcare provider
